# Zebrafish larva interface: an accessible, modular platform for *Danio rerio* experiments

**DOI:** 10.3389/fnins.2025.1593930

**Published:** 2025-06-30

**Authors:** John Jutoy, Hossein Mehrabi, Erica E. Jung

**Affiliations:** Department of Mechanical and Industrial Engineering, University of Illinois at Chicago, Chicago, IL, United States

**Keywords:** zebrafish larvae, *Danio rerio*, optokinetic response (OKR), biomachine, closed loop system, open-source, machine interface

## Abstract

The optokinetic response (OKR) in larval zebrafish (*Danio rerio*) is a well-characterized visuomotor reflex used to investigate sensorimotor integration. Building on prior work, we introduce the zebrafish larvae interface (ZLI) platform, a modular and accessible framework that enables closed-loop neuro-robotic experiments. We investigated how larval OKR behavior can translate to dynamic motion control of a wheeled robot. The platform incorporates an agarose stamping methodology to head-fix a larva while preserving full ocular mobility and visual access. Eye movements are recorded in real time using either a low-cost webcam or a microscope camera and processed through open-source computer vision software, which extracts eye angles via ellipse fitting. These measurements are translated into movement commands for a robot navigating a line-following task. The robot’s positional deviation is simultaneously converted into dynamic OKR-compatible visual stimuli displayed on an LCD screen beneath the larva, thus completing the sensorimotor loop. We demonstrate that the ZLI system enables larvae to robustly correct robot trajectories after substantial initial misalignment. By emphasizing modularity, affordability, and replicability, the ZLI system aims to democratize access to closed-loop behavioral research and promote widespread adoption in both educational and experimental neuroscience environments.

## 1 Introduction

Vision systems have been honed through millennia of natural selection, as vision is vital for motile organisms of the animal kingdom. Gaze stabilization is an adaptation of the visual system important for prey tracking and predator avoidance. Vertebrates employ two primary gaze stabilization mechanisms: the optomotor response (OMR), which involves body reorientation, and the optokinetic response (OKR), which involves compensatory eye movements in response to wide-field motion to reduce motion blur. Zebrafish (*Danio rerio*) have become a prominent model organism for studying visual processing due to their rapid development, transparent larvae, and conservation of vertebrate visual circuits (Bilotta and Saszik, 2001). Additionally, OKR has been observed in zebrafish larvae as early as 3 days post fertilization (DPF) (Easter and Nicola, 1996).

As zebrafish research has expanded into fields such as genetics, disease modeling, and optogenetics (Best and Alderton, 2008; Choi et al., 2021; Simmich et al., 2012; Portugues et al., 2013; Pérez-Schuster et al., 2016; Asakawa et al., 2021; Qian et al., 2023), there has been a corresponding rise in demand for tools that can reliably elicit and quantify visual behaviors like OKR. Traditional platforms, from Clark’s original rotating drums (Clark, 1981) to modern LCD-based stimulus systems (Rodwell et al., 2023), have advanced steadily. Some recent studies have employed innovative cost-saving approaches such as flip-phones or 3D printed components (Rodwell et al., 2024; Hermans et al., 2024; Gómez Sánchez et al., 2022), underscoring a collective push toward accessibility.

While these tools have expanded access to zebrafish visual research, most platforms remain fundamentally open-loop: visual stimuli are presented and the larva’s response is measured, but the animal’s behavior does not influence its sensory environment. Closed-loop paradigms—where an animal’s output dynamically modifies the input it receives—remain rare in larval zebrafish research. A notable exception is the work by Jouary et al. (2016), who developed a virtual reality setup where fictive swimming signals from paralyzed larvae updated visual stimuli in real time. However, such systems rely on immobilized preparations and virtual feedback.

Neuroethological studies have shown that zebrafish larvae dynamically adjust behavior based on visual context, particularly in tasks such as prey capture and optomotor stabilization (Bianco and Engert, 2015; Portugues and Engert, 2011). These works highlight the importance of studying sensorimotor transformations within ecologically meaningful feedback loops. However, such paradigms typically involve either freely swimming animals in open-loop visual environments or partially closed-loop virtual systems. Here, we present a system in which freely observing zebrafish larvae behaviorally control a physical robot, which in turn modulates their visual input. This setup enables a novel class of embodied closed-loop experiments, linking neural output to behavioral consequence and sensory feedback in a continuous, biologically grounded loop. In doing so, it extends the scope of neuroethological investigation to programmable and physically interactive environments—bridging reflexive behavior and adaptive feedback processing in real time.

Our approach builds on early foundational efforts in animal-machine interfacing, such as Reger et al. (2000), who demonstrated that lamprey brainstem tissue could drive a mobile robot in a bidirectional loop, with neural activity controlling motion and sensory feedback modulating neural output. In contrast, we employ intact, *in vivo* zebrafish larvae and allow their natural behavioral outputs—eye movements—to guide robot behavior. The robot then alters the visual stimulus experienced by the larva, completing a biologically embedded sensorimotor feedback loop. This represents a shift toward more ethologically relevant, perception-driven closed-loop experimentation.

To support this interaction, we introduce the zebrafish larvae interface (ZLI): a modular, low-cost platform that integrates larva fixation, visual stimulus presentation, and real-time eye tracking using accessible hardware and open-source computer vision tools. Larvae are immobilized via a stamped agarose cavity, eliminating the need for agarose sculpting or methylcellulose. The system is adaptable to other behaviors (e.g., tail, heart, or mouth movement) and developmental stages, making it a versatile tool for neurobehavioral studies.

We validate the ZLI by confirming its ability to elicit OKR responses using standardized metrics from Rodwell et al. (2023) in an open-loop configuration. To demonstrate its full potential, we also tested the system in a closed-loop configuration by interfacing the larva with a mobile robot. Eye movement signals, extracted in real time, were sent to the robot as control commands. In return, the robot’s position relative to a line was detected by an onboard camera and fed back to the stimulus module, dynamically adjusting the visual environment experienced by the larva. This implementation transforms the ZLI into more than just a visual stimulation and tracking platform—it becomes a biological control unit, where an intact nervous system governs and responds to its external context through robotic embodiment.

This approach offers significant benefits to the field. First, it provides a real-time, embodied readout of neural activity through behavior, effectively allowing the nervous system of a larval zebrafish to interact dynamically with its environment via a proxy body. This opens new avenues for studying sensorimotor integration, adaptive reflex modulation, and feedback-driven plasticity. For instance, researchers can examine how reflexive behaviors such as the OKR are altered under novel feedback contingencies, explore lateralization biases in visuomotor responses, or test how environmental changes influence temporal dynamics of reflex gain. Second, it offers a unique platform to investigate neuroethological dynamics in a controllable and reproducible setting, for example, how visual field geometry or motion parameters affect OKR strength and symmetry. Finally, the modularity and accessibility of the ZLI system enable researchers at various levels—from early-career students to expert labs—to engage with closed-loop biological interfacing without the financial or technical barriers traditionally associated with real-time experimental systems.

Our work echoes a conceptual legacy set forth by B. F. Skinner’s iconic “Pigeons in a Pelican” project, wherein pigeons were trained to steer a missile by pecking at targets on a screen (Skinner, 1960). While the system was ultimately never deployed, its influence was profound—demonstrating that biological organisms could be embedded into closed feedback loops to perform goal-directed tasks. That effort catalyzed new directions in behaviorism, control theory, and neuroethology. Similarly, our work shows that a larval zebrafish, using its optokinetic response, can control a robotic agent in real time—potentially enabling researchers to probe questions of behavioral asymmetry, adaptation, and innate preference. As with Skinner’s pigeons, our zebrafish may help reveal fundamental properties of biological intelligence, this time in a scalable, transparent vertebrate model.

Our platform unites neuroscience, robotics, and computer vision into a tractable, embodied, closed-loop system. It democratizes access to open-loop and complex feedback-loop experimentation for both researchers and educators, enabling new avenues of investigation into sensorimotor processing, lateralization, and biological control architectures in a powerful vertebrate model.

## 2 Materials and methods

### 2.1 Zebrafish larvae

All experiments with zebrafish larvae followed federal and local laws, with approval from the University of Illinois Animal Care Committee (ACC) and The Office of Animal Care and Institutional Biosafety (OACIB). Larvae were bred from wild-type adults in a temperature-controlled fish room (26°C) with a 14-h light and 10-h dark cycle. Eggs were harvested and maintained in the fish room until 4–5 days post-fertilization (dpf), then transferred to the testing facility the day prior to experimentation. During non-testing periods, larvae were kept in an incubation chamber maintained at 27°C.

### 2.2 Larvae fixation and imaging setup

**Figure 1:**
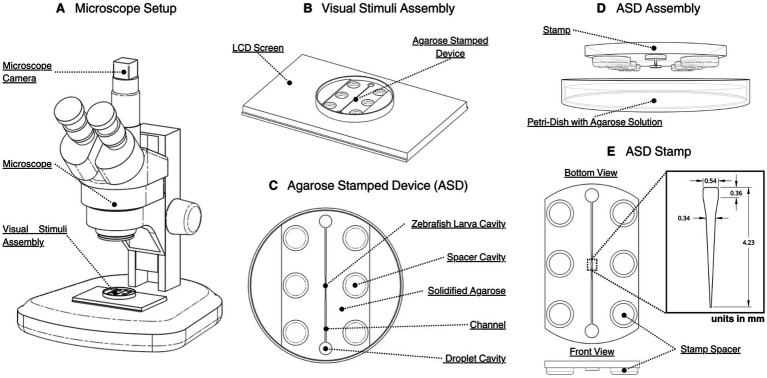
CAD renderings of the microscope-based tracking and stimulation system and the agarose stamped device (ASD) fixation system. **(A)** A stereo microscope fitted with a camera module, with the visual stimuli assembly mounted on the stage. **(B)** The visual stimuli assembly consisting of a 5-inch LCD screen with the ASD centered on its surface, enabling direct ventral side projection of images or animations to the larva. **(C)** A top view of the ASD consisting of a 50 mm petri dish filled with solidified 1.5% agarose with zebrafish larva cavities, spacer cavities, droplet cavities and the interconnecting fluidic channel created by the stamp. **(D)** Creation of the ASD assembly. The 3D-printed resin stamp positioned above the molten agarose in the petri dish, prior to pressing to form the cavities. **(E)** ASD stamp design. Bottom and front view of the resin stamp illustrating the six spacers that control larva cavity depth and other stamp features. Key dimensions of the larva outline positive on the stamp is provided (units in mm).

The microscope-based imaging setup, the visual stimulation assembly, and the agarose-stamped fixation system are the key components of the ZLI platform (Figure 1). In the microscope configuration (Figure 1A), an Amscope SM-1 Series stereomicroscope paired with a Ximea XiQ MQ013MG-ON-S7 camera provided high-resolution captures of larval eye movements. The visual stimulation assembly (Figure 1B) delivered rotating grating patterns to the larva’s ventral visual field via a 5″ capacitive LCD touch screen (Elecrow). The microscope setup is utilized for all experiments throughout this work.

The fixation system, referred to as an agarose stamped device (ASD) seen in Figure 1C, immobilized the larva in a larva shaped cavity in solidified agarose. The ASD was fabricated by pouring 1.5% agarose (Sigma-Aldrich A6877-100G) into a 47 mm Advantech PD-1 petri dish and pressing a 3D-printed resin stamp into the molten gel (Figure 1D). Once solidified (~10 min), the mold yielded a cavity matching the larva’s dorsal contour. The stamp (Figure 1E) incorporates six cylindrical spacers that ensure a consistent molding depth. These spacers leave cylindrical cavities that when filled with water, prevent agarose dehydration during extended (>15 min trials). Larvae are then gently slid into the cavity using a hair-loop device while excess fluid is removed and culminates with geometrically fixed larva. Excess water removal is important as larva could potentially dislodge itself from the cavity.

### 2.3 Visual stimulation module and eye tracking module

**Figure 2:**
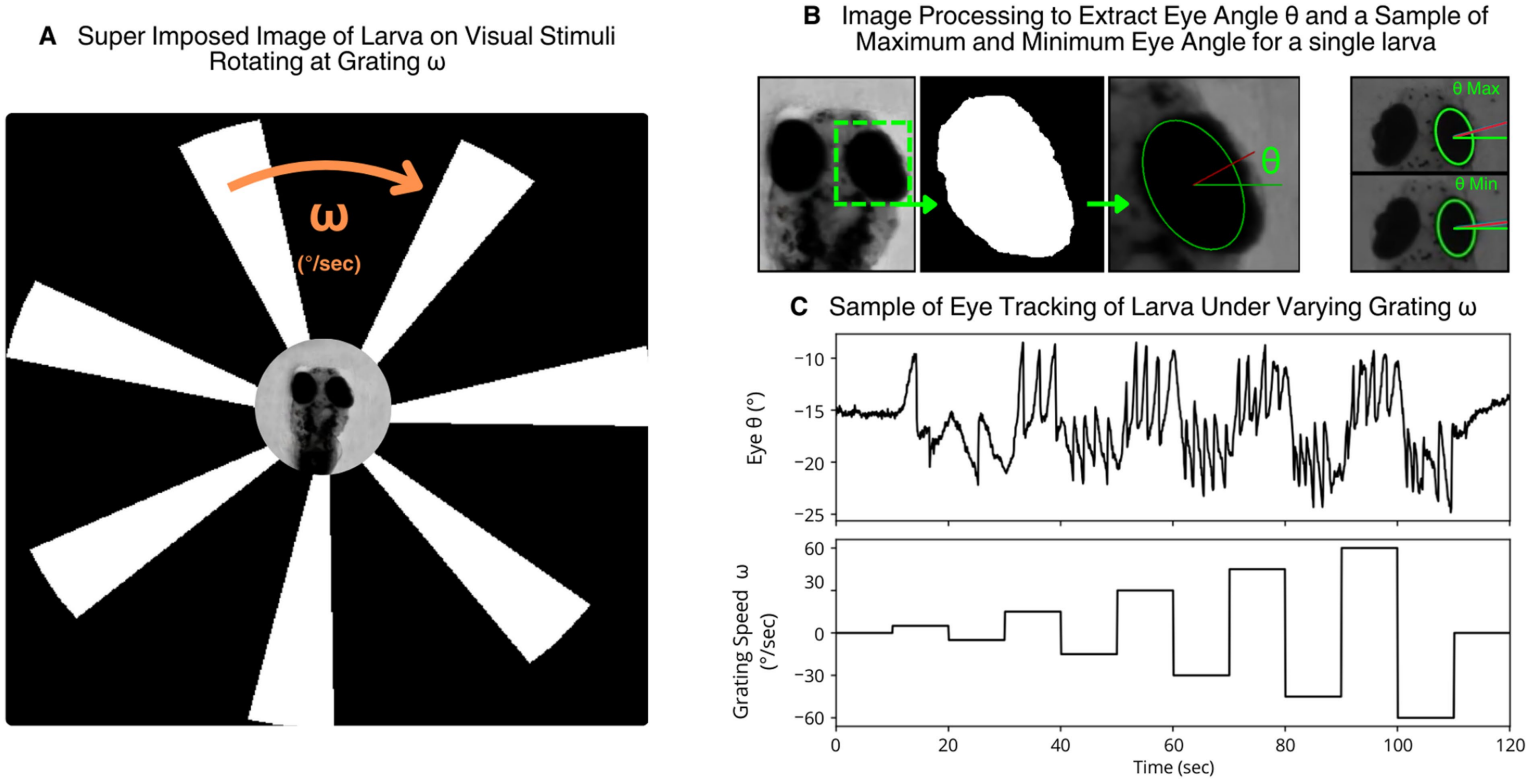
**(A)** Larva image from microscope camera superimposed onto the larva through an LCD Screen. The animation contains a circular set of black and white gratings at a rotational velocity 𝜔 that elicit OKR in the larva. **(B)** A zoomed in image of the larva in **(A)** with the region of interest (ROI) set on its right eye. Both eyes can be tracked simultaneously but only the right eye is displayed for simplification. The cropped ROI image passed through median blur and threshold filter. An ellipse is fitted to the contour and then superimposed to the eye of the larva. The eye angle *θ* is the angle between the minor axis and the horizontal axis. A sample maximum and minimum are presented to highlight larva position undergoing a saccade. **(C)** Plots of eye angle from **(B)** at changing grating *ω* over time.

A rotating grating animation was programmed in Python using OpenCV. This animation, displayed on the LCD screen beneath the larvae, consisted of concentric black- and-white gratings rotating around a central white contrast circle (Figure 2A). Parameters such as grating number, spacing, thickness, and rotational speed were real-time adjustable. The grating animation’s rotational velocity will be referred to as grating 𝜔 for simplicity.

Eye movement tracking was performed using custom software written in Python and OpenCV. Images were captured from the microscope camera and cropped to a region of interest (ROI) around the larval eyes. These images were converted to grayscale and filtered using a median blur and a threshold to isolate eye contours. Ellipses were then fitted to the contours, and the angle of the minor axis relative to the horizontal was defined as the eye angle (Figure 2B).

This angle was tracked over time to produce an eye movement time series (Figure 2C). Eye angle deltas (Δ*θ*) between successive frames were computed and used for further analysis, including identifying saccades.

### 2.4 Optimal threshold identification for signal filtering

An algorithm for finding the optimal threshold was developed to determine the threshold needed to reduce larvae eye signal noise that occurs from camera jitter. The method used to identify this threshold was to iterate over a set of possible threshold values with each iteration determining larva change in eye angle direction from frame to frame. Frame where the eye angle changes were in parity (aligned) with the direction of the animation were counted. A threshold that maximized the ratio between aligned frames versus misaligned frames was utilized for the line following trials. Any of the visual stimuli sets can be used with the threshold algorithm.

The result of a visual stimuli set yields a time series that is indexed from 0 to n , where n represents the final frame. From the resulting experimental time series, the deltas (difference between larva eye angle between consecutive frames starting at frame=2 , also equivalent to frame at time $t_{1}$ ) are first calculated for the angles of each larval eye ( θ ). The delta series of each eye ( Δθ ) are then parsed through the following threshold Equation 1.


(1) $$x_{\tau }(t)=\{\begin{array}{c} \mathrm{\Delta x}(t),\tau <0\\ \;\;\;0,\tau \geq 0 \end{array}$$

where x represents the left or right eye of the larva, and τ is the threshold value. The direction of the larva eyes (the sign of Δθ at time points $>t_{0}$ ), should have directional parity to the visual stimuli if the larva exhibits proper OKR. Therefore, a threshold value that maximizes the directional parity should be chosen. This was done through counting the frames of delta time series that were CCW (+) or CW (−) within a time bin (starting at $t_{b}$ and ending at $t_{\mathrm{bn}}$ ) and calculating the ratio between directional alignment of the larvae eyes to the visual stimuli. S of Equation 2 defines this ratio with its superscript denoting the visual stimuli direction and is a function of τ .


(2) $$S^{+}(\tau )=\frac{\sum_{t=t_{b}}^{t_{\mathrm{bn}}}P(\Delta \theta_{\tau })}{\sum_{t=t_{b}}^{t_{\mathrm{bn}}}N(\Delta \theta_{\tau })}\;\text{or}\;S^{-}(\tau )=\frac{\sum_{t=t_{b}}^{t_{\mathrm{bn}}}N(\Delta \theta_{\tau })}{\sum_{t=t_{b}}^{t_{\mathrm{bn}}}P(\Delta \theta_{\tau })}$$

where: $P(y)=\{\begin{array}{c} 1,y>0\;\\ 0,y\leq 0 \end{array}$ and *N* $(y)=\{\begin{array}{c} 1,y<0\;\\ 0,y\geq 0 \end{array}$

This method differs from more conventional statistical approaches, such as those based on baseline noise distributions or the standard deviation of eye angles during non-stimulated periods. However, we found that our method directly linked threshold values to task-relevant signal alignment and provided intuitive, behaviorally grounded cutoffs. A discussion of this methodological choice and its implications is provided in the Discussion section.

### 2.5 Experimental module and OKR animation characterization

An experiment coordination module was developed to synchronize visual stimulus changes and eye tracking. It accepted parameter schedules and recorded both eye angles and animation parameters every 0.1 s.

**Figure 3:**
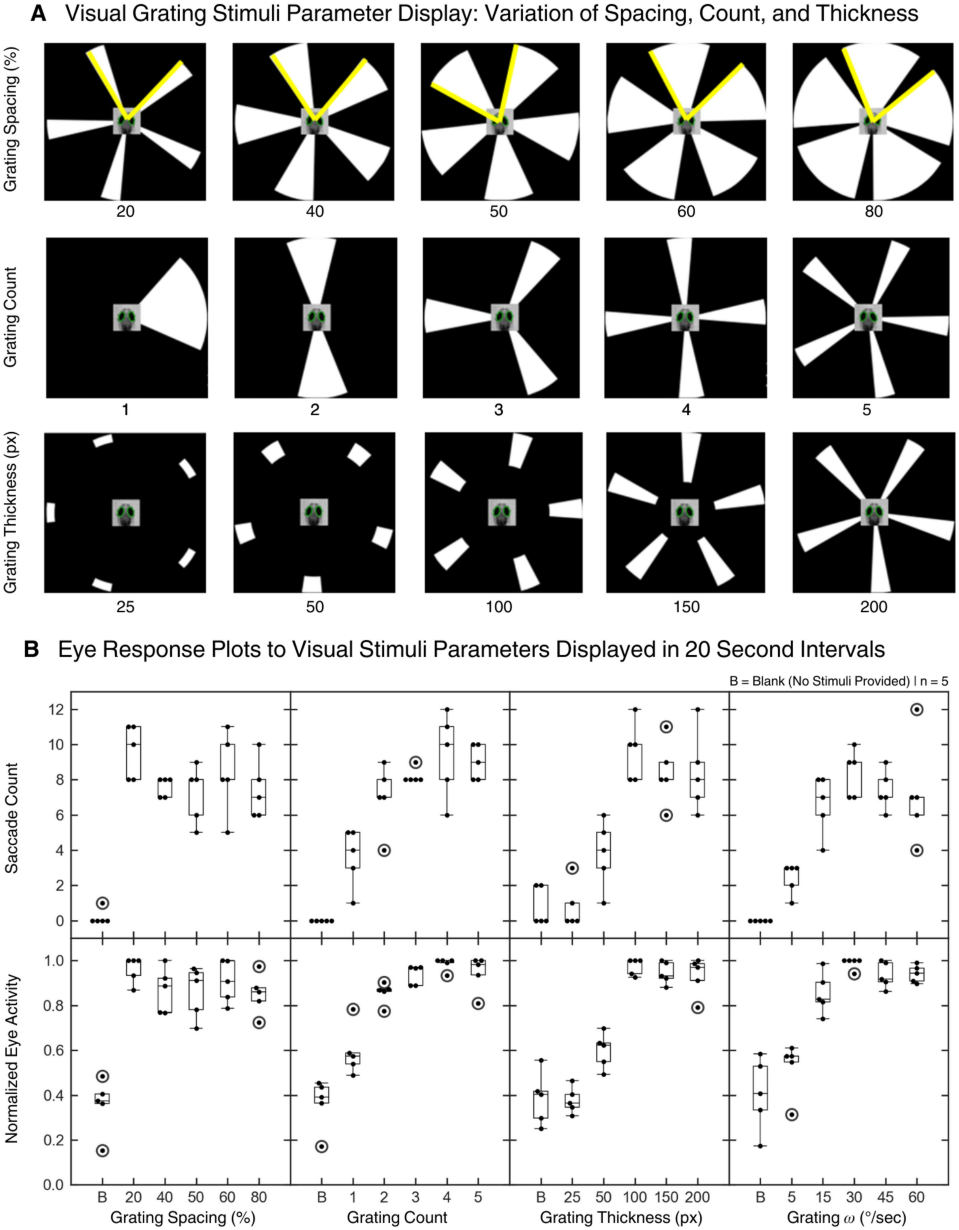
Characterization of optokinetic response visual stimuli parameters through parameter variation over time trials. **(A)** Visual stimuli parameters: grating spacing, number of gratings, grating thickness with larva superimposed in the middle. A trial set for a single fish consists of varying a single parameter over 2 min, 100 sec for each of the visual parameters explored. Each stimuli variation had a duration of 20 s and occurred sequentially. **(B)** Box plots of saccades and normalized eye activity (NEA) resulting from visual stimuli trial sets displayed in **(A)** and speed trial varying grating angular velocity 𝜔 (Figure 2C) on larvae at 7 days post fertilization ( n=5 ). Saccades and NEA of the left eye were extracted from the eye angle time series from each larva for each stimuli test. Each trial time series was binned to their corresponding stimulus. Peak thresholding of eye velocity was used to quantify the saccades. For the NEA, the sum of the eye angle difference from frame to frame within a stimuli bin was calculated and normalized to the bin resulting in the greatest sum. Time bin B represents eye activity during the time where a blank stimulus was provided, the first and last 10 sec of every stimulus trial. Large circles represent outliers with values 1.5 times greater or less than the upper and lower quantiles, respectively. With the NEA results, blank stimuli were found to be statistically significant with respect to at least one un-blanked bin for every stimulus parameter (Bonferroni-adjusted *p*-values ranging from: 0.0007–0.0374).

With the experiment module, further characterization was done for grating spacing, number of gratings, and grating thickness (Figure 3A). Each visual parameter is displayed in a 2-min trial with their corresponding changes occurring at t = 10, 30, 50, 70, and 90 sec respectively. No visual stimuli are displayed in the first and last 10 sec of the trial. For each visual parameter trial, the parameters not being varied were kept at: rotational speed = $30^{{}^{\circ}}/\sec$ , grating spacing = 20%, grating thickness = maximum, number of gratings = 5. Every 10 s the direction of rotation was alternated.

Five larvae were processed through a set of visual parameter trials. Each set consisted of a trial varying grating 𝜔 from Figure 2C, and the grating spacing, grating thickness, and number of gratings from Figure 3A. The resulting parameter and eye movement datasets were parsed to identify saccades and the normalized eye activity (NEA) of the left eyes of the larvae (Figure 3B).

Saccades were defined to be values greater than a threshold determined by eye and stimuli directional disagreement. A script was developed to iterate over a set of threshold values and was applied to the eye angle difference (eye deltas) between each time step for every trial. Each threshold iteration yielded the ratio of eye delta stimuli disagreements to agreement. The threshold that maximized the disagreement to agreement ratio was chosen to be the threshold value for saccade identification.

We define the NEA in Equation 3 as the summation of the absolute, frame-to-frame changes in eye angle within each time bin normalized to the bin with largest summed delta for that larva under each stimulus condition.


(3) $$\;\mathrm{Let}\;B_{i,t,b}=\sum_{f_{b,\text{start}}}^{f_{b,\mathrm{end}}-1}|\theta_{f+1}-\theta_{f}|,{\mathrm{NEA}}_{i,t,b}=\frac{B_{i,t,b}}{\max_{b\prime }(B_{i,t,b\prime })}\;$$

where i indexes the larva, t the trial stimulus condition, and b the time bin. $B_{i,t,b}$ is the total absolute change in eye angle within bin b . Finally, $\max_{b\prime }(B_{i,t,b\prime })$ represents the largest B over all bins b′ for that larva i and trial t .

For each stimulus parameter (width, grating spacing, thickness, speed), NEA values from the six experimental bins were pooled and ranked. We then applied the Kruskal–Wallis *H* test to these ranked NEA data to determine whether at least one level differed in median NEA. Following a significant global result, we performed Dunn’s pairwise comparisons on NEA ranks, adjusting all *p*-values with the Bonferroni method to maintain *α* = 0.05 for the family of contrasts. All computations were executed in Python (SciPy v1.9.3 for Kruskal–Wallis; scikit-posthocs v0.6.7 for Dunn’s), and reported *p*-values reflect Bonferroni adjustment.

### 2.6 Alternate eye tracking setup: webcam

**Figure 4:**
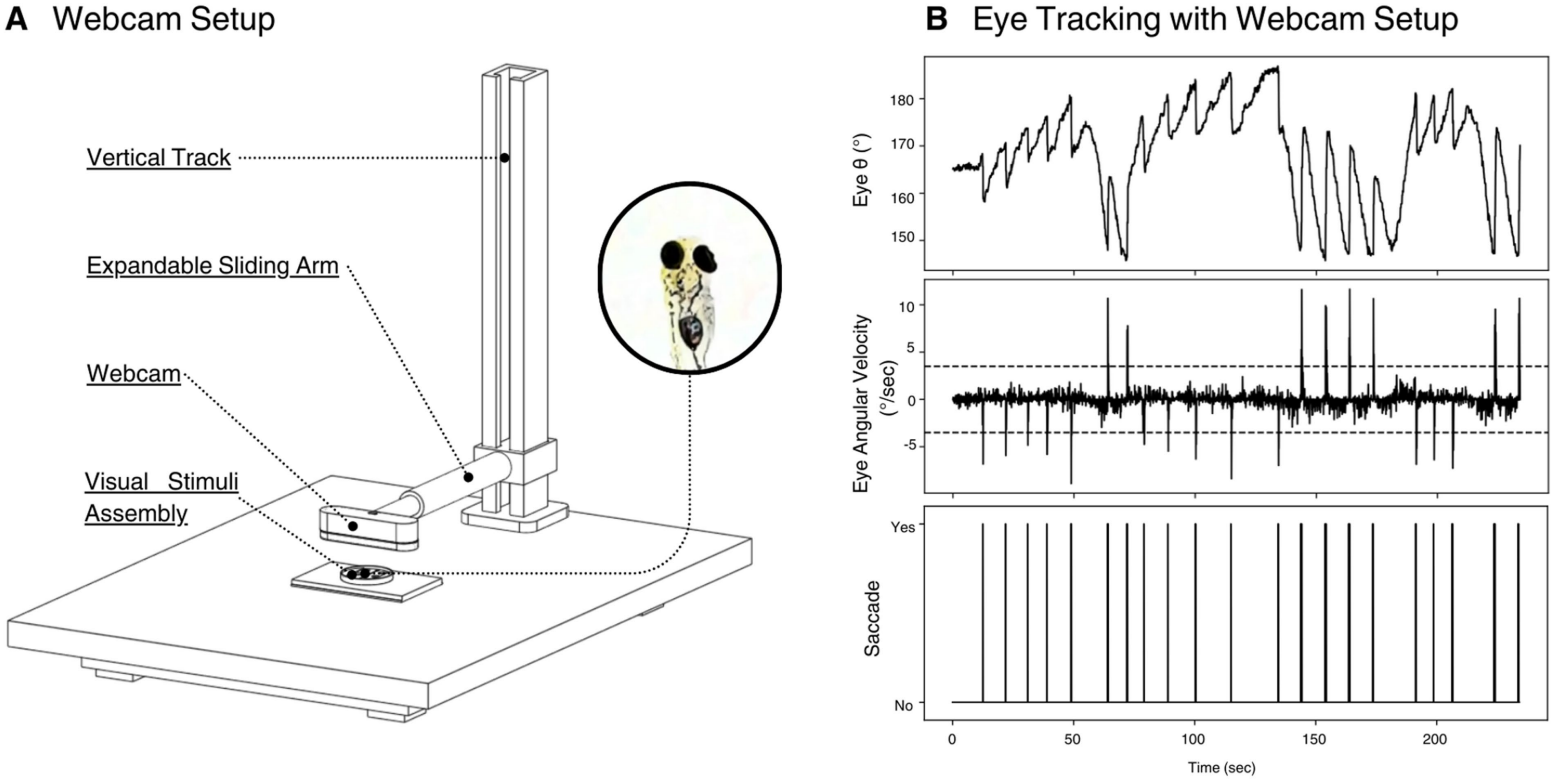
Webcam-based eye-tracking setup and representative data. **(A)** Schematic of the consumer-grade webcam configuration. A 4K-resolution webcam is mounted on an expandable sliding arm affixed to a vertical track, both anchored to an optical breadboard. This arrangement allows fine adjustment of camera height and focus. The webcam is aimed at the visual stimulus assembly from Figure 1B. **(B)** Representative output from a single 240-s trial using the webcam setup. Top panel shows raw eye angular position *θ* in degrees as a function of time. Middle panel plots the instantaneous eye angular velocity (°/sec), with dashed lines marking the ±3°/s thresholds used for saccade detection. Bottom panel displays the resulting binary saccade events (“Yes” = detected, “No” = none) aligned on the same time axis.

To evaluate a low-cost, flexible alternative to our microscope configuration, we included a webcam-based setup for larval eye imaging (Figure 4A). A consumer-grade 4K webcam (Logitech Brio; Webcam) was mounted on an expandable sliding arm attached to a vertical aluminum track, all secured to a vibration-isolated optical breadboard. The visual stimulus assembly from Figure 1B is rested centrally on the breadboard directly beneath the camera. The webcam lens was positioned ~5 cm above the specimen, allowing for high framerate low resolution and low framerate high resolution recordings.

We then validated this setup by presenting a pseudo-random rotational stimulus via our custom visual stimulation system. Eye angles were extracted with our Python tracking software, and the eye angular velocity, and saccades were extrapolated from that data. The time-series plots in Figure 4B were developed for analysis.

### 2.7 Secure shell and robot interface module

A secure shell (SSH) module built on Python Paramiko enabled communication between the workstation and a Raspberry Pi-controlled three-wheeled robot. Robot-side modules included movement control (via RPI.GPIO), line identification, and I/O listeners for SSH. The SSH module initiated scripts on the robot, collected line center-of-mass data, and sent movement commands.

The robot is constituted of a Raspberry Pi (Raspberry Pi 4 Model B), a Raspberry Pi camera (Rev 1.3), two motored wheels (DC Gearbox Motor 200 RPM 3-6VDC) controlled by a motor driver (L298N), a castor wheel, a portable battery for the Pi, a battery for the motors, off the shelf chassis, and various 3D printed parts. A concept bill of materials is included in the Supplementary material.

### 2.8 Line following experiments

**Figure 5:**
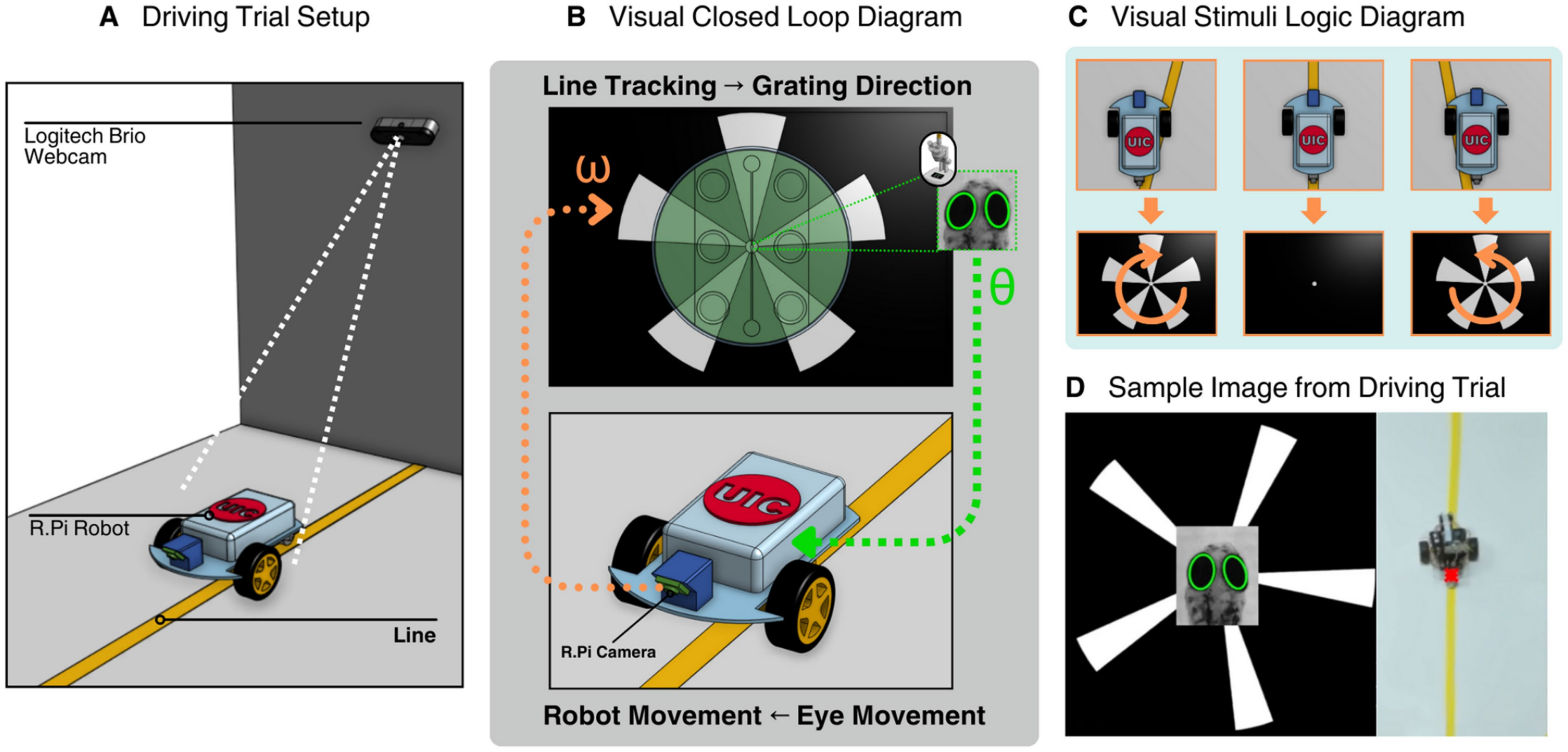
System setup of zebrafish larva interface including line following robot setup, closed loop system, animation display logic, and a representative image of a driving trial. **(A)** Rendering of the robot during a closed loop trial run, a Logitech Brio Webcam tracks the position of a point on the rear of the cam with the line in frame. **(B)** Rendering of the closed loop system displaying a larva inside the visual stimulation assembly within the microscope setup interface with the robot. The larval eye movements are translated to robot movements and the onboard camera from the robot identifies its location with respect to the line and sends a signal to the visual stimulation module to change the displayed animation. **(C)** A visual diagram of the animation display logic. Clockwise grating rotation is displayed if the robot identifies it is on the left side of the line, hides the gratings if it is directly on the line and counterclockwise if the robot is on the right side of the line. **(D)** A representative image from a driving trial experiment consisting of the visual stimulation animation, zebrafish larva eye tracking, and robot positional tracking.

To test the larval and robot interface, an experiment was designed to see if larval optokinetic response was able to keep a robot on a line (Figure 5A). Larva controlled the robot by modulating eye angles in response to grating direction. Simultaneously, robot perception of its location relative to the line fed back into the visual stimulus loop (Figure 5B).

The track was constructed from white poster paper (55 cm × 55 cm) taped to the floor, with a 2.54 cm wide strip of yellow painter’s tape running through the center as the line to follow. The robot’s onboard camera used HSV filtering to isolate the line based on color, and the center of mass was calculated for navigation.

An initial speed characterization trial was used to compute individualized eye angle thresholds that filter out jitter from camera or larval micro-movements. The optimal threshold identification algorithm was iterated within the limit range of τ=[1,55] . The threshold corresponding to the maximum ratio is used throughout the duration of line following trials for a single larva. This allows the eye tracking system to send the best signal of larva eye rotation to the robot.

The robot was set to move forward if no eye movement was detected. To do this, both eyes ran through the identified threshold in real time from consecutive frames to determine the signal sent to the robot (Equation 4). Only when both eyes are both greater or less than their respective thresholds is a turn signal sent. The previous work finding the thresholds τ for both eyes was used to filter out camera noise that could mask low Δθ values.


(4) $$\text{Movement}(\Delta \theta )=\{\begin{array}{c} \text{Left},\Delta \theta_{L,R}>\tau_{L,R}\\ \text{Right},\Delta \theta_{L,R}<\tau_{L,R}\\ \text{Forward},\text{Otherwise}\; \end{array}$$

where:


$$\Delta \theta_{L,R}>\tau_{L,R}\to \Delta \theta_{L}>\tau_{L}\;\text{and}\;\Delta \theta_{R}>\tau_{R}$$

$$\Delta \theta_{L,R}<\tau_{L,R}\to \Delta \theta_{L}<-\tau_{L}\;\text{and}\;\Delta \theta_{R}<-\tau_{R}$$

The grating display was determined by the robot’s identification of where it was with respect to the line. If the line was centered, gratings were hidden. If off-center, the animation rotated CW or CCW accordingly (Figure 5C). Each larva completed eight trials (4 speeds × 2 directions), and all positional and angle data were recorded at 10 Hz.

A Logitech Brio webcam mounted 7 ft. above the track area captured the robot’s position A colored marker at the robot’s rear enabled tracking. Images were perspective-corrected in real-time using the cv2.warpPerspective function to simulate a top-down view. Robot position was tracked via color thresholding and contour center-of-mass detection (Figure 5D).

### 2.9 Summary of trial structure

Each line-following trial began with the robot at rest, offset at an oblique angle to the track. Upon initiation, the larva received visual stimuli, and its eye movements dictated robot direction. The cycle continued for 45 s per trial. Trials were conducted between 3:00 PM and 9:00 PM.

Data outputs included timestamped CSV files containing robot coordinates, eye angles, and visual stimulus parameters. For error analysis, the absolute x-error (deviation from the track center) was computed and plotted across time. Additional metrics such as intersection time and cumulative error were used to evaluate trial performance.

Each larva completed the full set of conditions within 13–15 min barring technical issues, with longer durations (up to 30 min) required when Raspberry Pi communication errors occurred.

## 3 Results

### 3.1 Validation of eye tracking and visual stimulation system

Using the developed software and hardware modules, we validated the system’s ability to elicit and track optokinetic responses (OKRs) in larval zebrafish. Eye angles were successfully extracted and visualized in real-time as larvae responded to ventrally displayed rotating grating animations (Figures 2C, 4B). Eye movement traces showed clear modulation in response to changes in stimulus direction and speed, supporting the efficacy of the custom tracking and stimulation setup.

### 3.2 Stimulus parameter characterization

To characterize how different visual stimulus parameters influence OKR, we systematically varied grating rotational velocity 𝜔, spacing, thickness, and number of gratings. Across five larvae, we observed robust OKRs with predictable changes in eye movement patterns. Analysis of saccadic activity revealed distinct thresholds for stimulus-driven movement, and the normalized eye activity (NEA) metric provided a comparative measure across trials (Figure 3B). The results confirmed that the grating animation resulted eye movement compared to when it is not displayed.

### 3.3 Closed-loop neuro-robotic interface: larva-driven robot trials

We implemented a closed-loop system wherein larval eye movements directly controlled the direction of a Raspberry Pi-based robot. The robot’s behavior was simultaneously used to adjust visual stimuli, creating a bi-directional feedback loop (Figure 5B).

**Figure 6:**
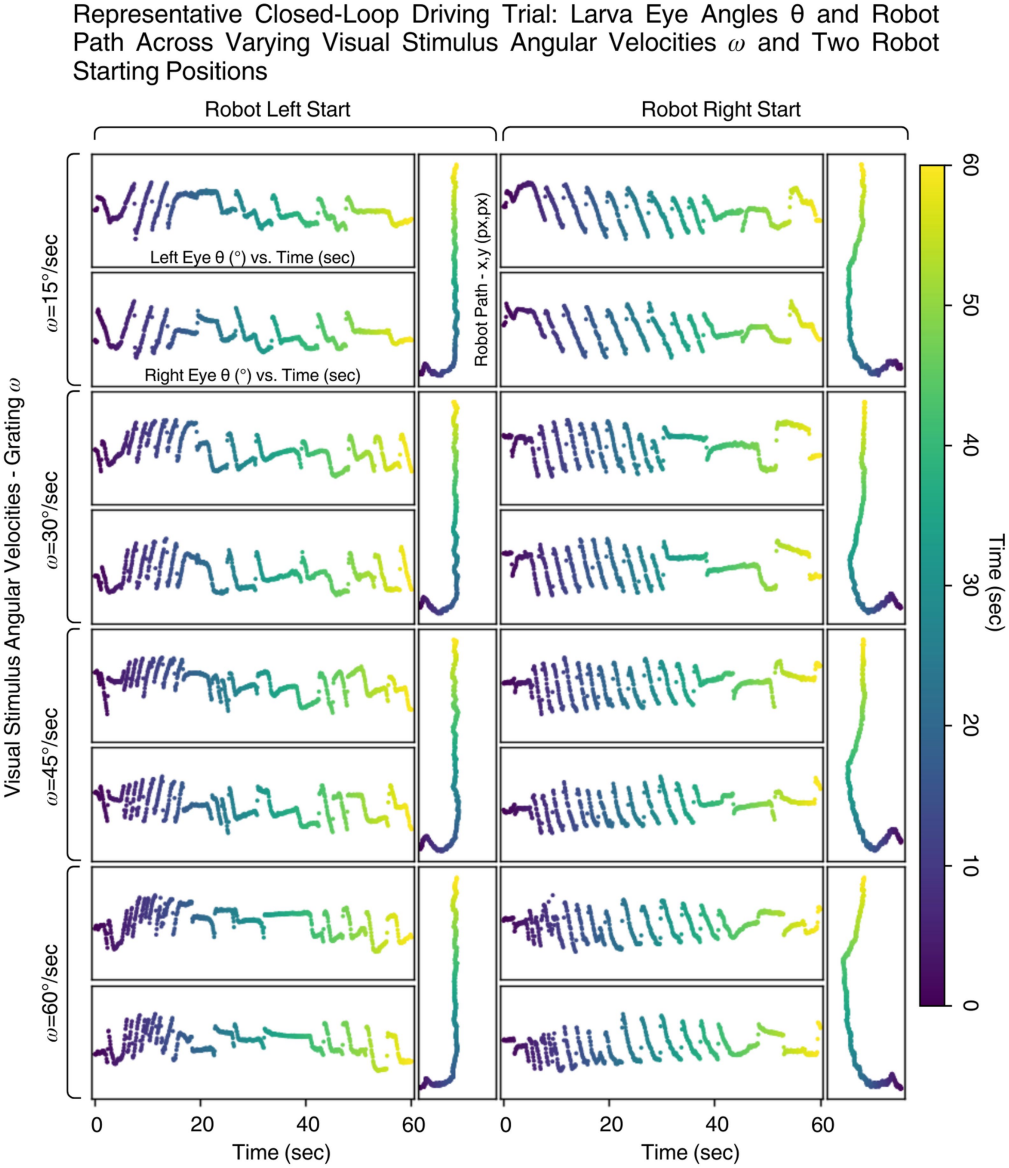
Closed loop driving trial array for a single larva at different robot starting positions—left or right side of the yellow line seen in Figure 5 at different grating angular velocities ω (15, 30, 45, 60°/sec. Each pair of grating ω and robot starting position consists of plots of the larva’s left and right eye angles *θ* over time along with the respective plot of the robot path. Time is mapped through gradient color between the eye angle plots and the robot path plot.

In driving trials, larvae were tasked with keeping the robot aligned to a visual line using their OKR. Across various rotational speed conditions (15–60°/sec), larvae successfully drove the robot to re-align to the line after an initial misalignment (Figure 6). The eye movements consistently triggered correct directional commands, resulting in forward, left, or right turns by the robot based on real-time signal thresholds derived from previous calibration trials.

### 3.4 Performance metrics from driving trials

**Figure 7:**
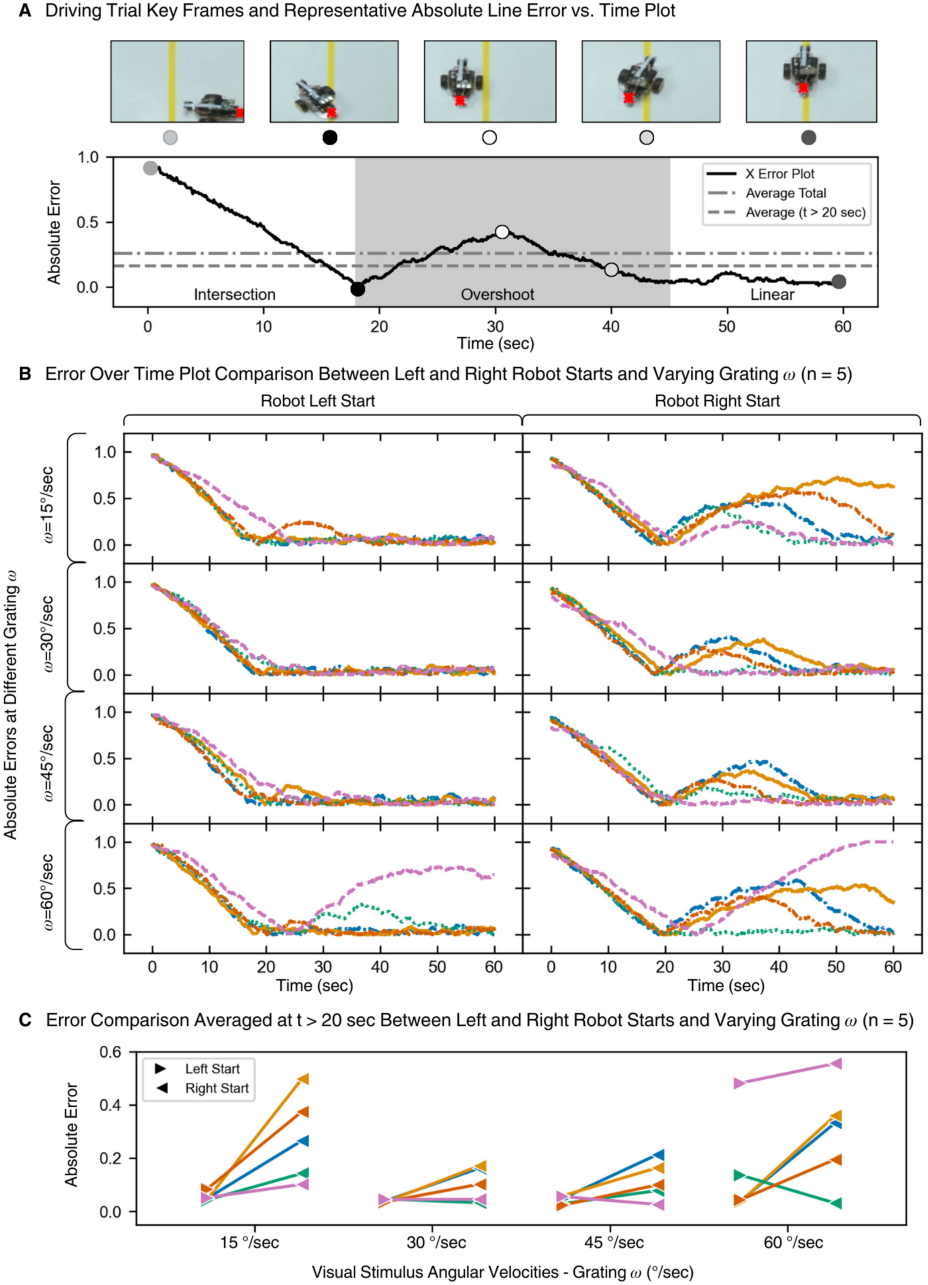
Analysis of closed loop driving trials. **(A)** Key frames from the driving trial are marked on a representative plot of the absolute error (between car position, the red x, and yellow line) over time. Averaging of the total error and total error after intersection region (> 20 sec) are plotted to show closed loop performance. **(B)** Absolute error over time plots for n=5 larva doing the closed loop driving trial array. Each color corresponds to a single larva. **(C)** Average of absolute error time series data (*t* > 20 sec) for each stimuli angular speed ω to highlight the differences between left and right starts of the robot.

Quantitative analysis of robot trajectories demonstrated the closed-loop system’s effectiveness. The absolute x-error metric captured how far the robot deviated from the line at each timepoint. A characteristic overshoot followed by correction and steady line following was observed across trials (Figures 7A,B).

Cumulative performance across trials (*n* = 5 larvae × 8 trials) revealed consistent behavior: once the robot crossed the line, larvae were able to maintain alignment across varying speeds and initial directions (Figure 7C). Notably, robot performance appeared influenced by initial direction, a phenomenon explored further in the Discussion.

## 4 Discussion

### 4.1 Triggering optokinetic response with ventral LCD projection

Most traditional methods for evoking the optokinetic response (OKR) in zebrafish larvae rely on rotating drums or projected patterns that are oriented around the sides or front of the animal-typically stimulating the frontal-lateral visual field. More recently, studies have shifted toward using digital displays like LCD or LED panels to deliver visual stimuli (Dehmelt et al., 2018; Rodwell et al., 2024). However, these displays are still usually placed laterally or frontally and rarely target the underside of the larva.

In contrast, our study introduces a simple and accessible setup that uses a standard LCD screen placed directly beneath the larva to display a rotating circle animation composed of black and white gratings. This approach offers a minimal footprint, low-cost alternative to the more complex projection systems commonly used in OKR studies.

Ventral visual stimulation has been explored before, but usually with different goals and technologies. Portugues et al. (2014) projected wide-field motion across a light-diffusing screen that surrounded the larva, including from below, but did so as part of a whole-sphere setup designed for brain-wide calcium imaging—not for standalone behavioral assays. Similarly, some optomotor response (OMR) studies, such as those by LeFauve et al. (2021), have used LCD screens under the larva to present whole-field translational motion. However, these setups target OMR, which is a locomotor response, not OKR, which is a reflexive eye movement.

To our knowledge, no prior study has demonstrated clear and robust OKR elicitation in zebrafish larva using a discrete visual stimulus presented exclusively from the ventral plane using an LCD screen. Unlike projectors, which diffuse light broadly and often require alignment and calibration, our system delivers precise animations at very close range (~5 mm from the larval eye), enabling robust OKR without specialized optics or enclosures. This is notably closer than the typical 2–5 cm distance used in most OKR studies (e.g., Tuschl et al., 2022).

Despite this proximity and unconventional direction of stimulus, the larvae reliably tracked the rotating pattern. As shown in Figure 2C, the eye angle closely followed the motion of the stimulus and became erratic or flat (seen in the first and last 10 s of the eye angle over time graph) when the animation was paused or removed. Both slow and fast OKR phases were clearly visible, and the response remained strong even in low background ambient light, although it was slightly improved without background light present.

The underlying mechanism of OKR elicitation from below was not the focus of this study but may involve the larva directly detecting motion from beneath, similar to OMR-like behavior, or may result from refraction of the visual stimulus into the lateral view via the water-agarose-air interfaces. Regardless of the precise optical path, our findings show that robust OKR can be achieved with a ventral LCD-based setup—introducing a practical and replicable platform for studying zebrafish visual behavior in low-plane fields without the need for complex optics.

### 4.2 Accessible eye and visual stimulation experimental setup

To reduce the barrier of entry for laboratories with limited resources or technical expertise, we developed a modular experimental setup that is both adaptable and accessible. The system requires only four essential components: a display (LCD screen), a fixation platform, an imaging device (either a microscope or webcam), and a computer. This streamlined architecture contrasts with many traditional setups that depend on complex optomechanical assemblies or projector-based visual stimulation systems.

Two parallel configurations were designed to accommodate varying levels of laboratory resources and imaging resolution requirements (Figures 1, 4). The microscope-based setup allows for high-resolution eye tracking and fine-grained data collection, suitable for experiments demanding sub-pixel accuracy. In contrast, the webcam-based setup provides a more accessible and cost-efficient alternative, using a 4K consumer-grade webcam mounted on a simple vertical rail system. Despite its affordability, this configuration proved sufficient for capturing optokinetic eye responses and offers an excellent entry point for labs new to behavioral neuroscience or working with limited budgets.

To further increase throughput and simplify larva handling, we developed a novel fixation method inspired by Copper et al. (2018), who employed agarose stamping for histological preparation. We adapted their technique by engineering stamped agarose wells tailored to the size and morphology of 7 dpf zebrafish larvae. This design enables consistent dorsal-up orientation and gentle immobilization while preserving natural eye mobility-crucial for accurate OKR measurements. The transparent nature of both the agarose and petri dish ensures that stimuli presented on the underlying LCD remain clearly visible to the larvae.

The agarose stamped methodology avoids full embedding in liquid agarose, allowing for larva to be used for longitudinal studies without fear of impairing its ability to eat. Furthermore, no additional processing – like removal of excess agarose around the head—is required if free eye movement head fixation is desired. This method also eliminates any need for the popularly used low-melting-point (LMP) agarose, which is twice the cost of standard agarose.

This stamping approach simplifies and accelerates fixation since larva only contacts already solidified agarose. After each trial, a larva can be dislodged by pipetting water into the larva cavity. With proper handling, the device remains reusable for multiple larvae, provided it is kept hydrated. The stamps can be designed for high-throughput formats (e.g., arrays of 15 cavities per device). Additional details on concentration optimization, stamp design variations, and step-by-step protocol refinements are provided in a forthcoming manuscript (DOI: 10.1101/2025.03.04.641502).

Importantly, the system remains compatible with more conventional fixation media such as methyl cellulose or standard agarose embedding, maintaining methodological flexibility. By combining low-cost components with an innovative yet simple fixation strategy, this setup fosters reproducibility and scalability, enabling high-throughput visual neuroscience experimentation in a wide range of institutional settings.

Although our system was designed for 5–7 dpf larvae, its modular setup can be easily adapted for embryo to older larvae. By adjusting the fixation and tracking parameters, the same approach can be used to study different developmental stages. Beyond eye movements, the system can be modified to track other features like heart rate, mouth movement, or tail motion, making it a versatile tool for a wide range of behavioral studies.

### 4.3 Modular and replicable software framework

A core tenet of the system’s design philosophy is accessibility—not just in hardware, but in software architecture. To that end, all custom software developed for this project has been released as open source, accompanied by detailed documentation to encourage replication, modification, and extension by the broader research and educational communities.

At the heart of our system lies a modular framework built using Python and OpenCV. This framework is intentionally structured to mirror the physical modularity of the experimental setup, consisting of loosely coupled software components that handle visual stimulus generation, real-time video capture, feature tracking, and data communication. Each component can be reused or swapped out independently, making the system highly adaptable to different organisms, behaviors, or hardware setups.

We also provide a blueprint for designing and developing behavioral or closed-loop systems using Python-based computer vision. This includes:

- A stimuli presentation module, allowing for user-defined animations or patterns to be presented through accessible display hardware.
- A real-time tracking pipeline, utilizing OpenCV for image preprocessing, feature isolation, and geometric fitting (e.g., eye ellipse fitting).
- A communication layer, capable of transmitting signals (e.g., eye positions or decisions) to external hardware, such as robots or feedback controllers.

An experimental orchestration layer, which coordinates input, processing, and output in a manner flexible enough to support custom trial structures or feedback logic.

This design enables others—with some programming experience—to construct and deploy their own variants of the system. Importantly, Python’s readability and its expansive ecosystem of packages lower the barrier to entry for researchers, educators, and even undergraduate students to engage with or adapt our framework for their own use cases.

By sharing not just code but an architectural approach, we aim to support the broader adoption of open behavioral and neuroscientific tools that are affordable, flexible, and extensible. The code base, example configurations, and modular template scripts are available at https://github.com/JJutoy2/Zebrafish-Larva-Interface.

### 4.4 Eye tracking module validation

Consequently, since OKR was clearly observable in the time series, we can state that the developed eye tracking software is sufficient for eye tracking. This was expected as other developed software used similar tracking methodologies of image thresholding followed by ellipse fitting (Roeser and Baier, 2003). Although there are commercially developed stimulation and tracking systems like ZebEyeTrack (Dehmelt et al., 2018), DanioScope (DanioScope, n.d.), and VisioBox (VisioBox, n.d.) along with open sourced software and systems like ZebraZoom (Mirat et al., 2013), Stytra (Štih et al., 2019) (Python), BonZeb (Guilbeault et al., 2021) (C#/.NET), and (Matlab) (Scheetz et al., 2018), we developed our own low cost software/tracking platform in order to provide accessible options for other researchers. Additionally, creating our own platform made syncing easier with the visual stimulation module, robot module, and future modules we plan to develop.

Using an LCD screen and a custom-built animation software provided the benefit of easily customizable animations along with real-time adjustable and timed display experiments. This freedom allowed for the development of the closed-loop system discussed in the following section but also allowed for visual stimuli characterization. Timed tests were developed that changed a single parameter to determine how a visual parameter affects the larvae response. Particularly, we were interested in how speed, spatial frequency of gratings, and distance to grating affected the larva OKR response.

We chose to analyze the normalized eye activity (NEA) because it captures the overall magnitude of eye movements in a single continuous measure. Because each condition had only five observations and our NEA data did not meet the assumptions of a standard ANOVA, we used the Kruskal–Wallis *H* test, a rank-based method that does not require normality or equal variances. When this omnibus test showed a significant difference among levels (*α* = 0.05), we followed up with Dunn’s pairwise comparisons and applied a Bonferroni correction so that the chance of a false positive across all comparisons stayed at 5%. Blank stimuli were found to be statistically different (Bonferroni-adjusted *p* < 0.05) with at least one nonblank variation of a stimulus set: blank vs. 20% grating spacing (*p* = 0.00235), blank vs. 3, 4, 5 grating count (*p* = 0.0374, 0.0007, *p* = 0.0072 respectively). Blank vs. 100 px grating thickness (*p* = 0.0072), blank vs. 30°/sec grating angular velocity 𝜔: (*p* = 0.0011). However, no statistical significance was found from parameter increments. This may be due to the arbitrary choosing of increments for the parameters and the low sample count. The trials were also done sequentially instead of randomly which may elicit a bias. Regardless, at the very least, we can conclude that the larva OKR are elicited by our system.

### 4.5 Threshold methodology

In this study, we adopted a task-specific, data-driven approach to determine threshold values for detecting effective eye movements and saccades, aiming to directly optimize the alignment between larval eye responses and stimulus dynamics. While more conventional statistical methods, such as defining saccades using thresholds at ±3 standard deviations from baseline distribution, are commonly used in oculomotor studies, we found such approaches to be less effective in the context of our dynamic, closed-loop paradigm. Specifically, periods of “no stimulation” still yielded subtle visual input due to screen luminance and prior stimulus memory, making them unreliable for establishing a clean baseline distribution. In contrast, our alignment-based method offered a behaviorally grounded and interpretable threshold that linked neural output to task-relevant sensory cues. Nonetheless, we acknowledge the value of complementary statistical approaches and encourage future comparative studies to explore whether hybrid models may further improve saccade and signal classification in similar real-time neuro-robotic systems.

### 4.6 Embodied readout and control: mapping OKR to a line-following task

To explore whether reflexive neural behavior like the optokinetic response (OKR) could be used to control a machine in real time, we integrated a mobile robot into our experimental setup (Figure 5A). In this system, the robot’s movement is driven directly by the larva’s eye movements, which are triggered by visual stimuli (Figure 5B). This setup transforms the larva’s internal neural activity into a physical action in the world, effectively making the robot a live readout of the larva’s behavior.

We applied this system to a line-following task, a common challenge in robotics that typically requires some form of self-correction. The goal was to determine if the larva, through its natural OKR reflex, could guide the robot along a visual path without any machine learning or engineered control algorithms.

Despite initial misalignments and physical imperfections in the robot’s motion (such as mechanical bias from the castor wheel), multiple larvae were able to consistently steer the robot toward and along a linear trajectory. As shown in Figure 6, the paths of the robot often converged to a straight or nearly parallel alignment with the target line. This outcome suggests that the reflexive behavior of the larva is not only responsive but also robust enough to overcome real-world noise and disturbances.

Importantly, this demonstrates more than just biological fidelity—it highlights a novel form of biological control. By embedding a simple organism into a feedback loop with a mechanical agent, we show that the nervous system of a larva can interface directly with and adapt to an external system. The larva acts as a kind of natural controller, responding to sensor input (visual stimuli) and producing motor commands (eye movements) that are mapped to the robot’s motion.

This has several implications. First, it presents a new way to study how reflexive neural circuits interact with dynamic environments. Second, it reduces the need for hand-designed models of noise, dynamics, or task-specific control algorithms. The only requirements are a known mapping between the larva’s eye movement and the machine’s actuators, and a way to feed the robot’s sensory data back into the larva’s visual field—conditions that are relatively simple to satisfy.

Finally, by converting internal brain activity into visible and measurable robot behavior, the system becomes highly accessible. The robot’s motion serves as an intuitive readout of the larva’s computation, making it easier for observers—even those without technical training—to understand what the system is doing in real time. This opens possibilities not only for scientific research but also for teaching, outreach, and prototyping hybrid bio-machine systems.

In sum, mapping OKR to a line-following task exemplifies the power of embodied readouts. It shows how a simple animal reflex can be leveraged for control, exploration, and interaction with the physical world, bridging neuroscience, robotics, and system identification in a single, modular platform.

### 4.7 Directional eye movement bias in larvae

Interestingly, a clear directional bias was displayed among the larvae during the driving trials. We found that all larvae exhibited better performance when the robot started from the left side of the line. Since overshoot regions were the source of navigational errors, the visual stimuli direction dominant in that region is critical to understanding the bias. Counterclockwise stimuli, dominant when starting from the left, elicited stronger OKR responses, allowing the robot to more reliably align with the line.

Previous works have observed asymmetric OKR responses in larvae, particularly showing higher OKR amplitude when stimuli move from temporal to nasal (T-N) across an eye (Roeser and Baier, 2003; Qian et al., 2005) Applying this known T-N asymmetry to our setup would suggest that the right eye should show greater response to counterclockwise stimuli. However, because our system averages angle changes from both eyes to drive the robot, any individual eye asymmetry should cancel out. Thus, if only T-N asymmetry were present, performance should have been equivalent regardless of the robot’s starting side—yet we consistently observed better performance when starting on the left.

This discrepancy suggests an additional bias beyond simple T-N asymmetry. We propose that larvae may possess an intrinsic directional preference in eye movement, independent of the T-N dominance, favoring a particular rotational direction. This is evident in the eye angle traces for a single larva undergoing multiple trials seen in Figure 6: when the robot started on the left, significant eye activity occurred between the 5–20 s mark for both eyes, whereas starting on the right led to delayed, more spaced-out eye activity, with the robot reaching the linear region only after 30–40 s.

Behaviorally, such a directional preference could be beneficial for schooling fish, where coordinated movement is critical. Social lateralization—genetically driven alignment of behavior across a group—has been documented in zebrafish and other species (Gebhardt et al., 2013; Bisazza, 1996). Moreover, studies like Barth et al. (2005) show that genetic mechanisms can coordinate the laterality of internal organs, brain asymmetry, and behavior in zebrafish. Thus, the directional eye movement bias observed in our experiments may reflect a socially advantageous form of lateralization that promotes synchronized group behavior.

## 5 Conclusion

In this study, we introduced the zebrafish larvae interface (ZLI), a platform capable of simultaneously delivering visual stimuli and recording behavioral responses in larval zebrafish. Central to our design is a novel yet simple fixation method using stamped agarose cavities, which effectively immobilizes the larvae without impeding visual input or eye movement output. We validated the system using the well-characterized optokinetic response (OKR), implementing customizable visual assays to identify parameters that robustly elicit OKR behavior.

With these optimal parameters, we extended the platform to include a robotic readout module, enabling closed-loop experiments in which larval OKR was used to control a line-following robot. This demonstrated both the flexibility of the ZLI and the feasibility of using OKR as a real-time biological control signal. Interestingly, these experiments also revealed directional bias in visual preference among larvae. While prior research has documented temporal-to-nasal asymmetries at the level of individual eyes, we propose that the observed bias may instead reflect a form of social lateralization—potentially linking the well-known turning bias in zebrafish to a shared visual attention bias.

Taken together, our work demonstrates that high-quality, closed-loop neurobehavioral experiments can be performed with accessible, off-the-shelf hardware and open-source software. By lowering technical and financial barriers, the ZLI aims to broaden participation in zebrafish research and inspire new investigations in neuroethology, behavior, and real-time neural control systems.

## Data Availability

The datasets, raw videos, code, and other supplementary information for this study can be found in the zebrafish-larva-interface GitHub repository: https://github.com/JJutoy2/Zebrafish-Larva-Interface.
